# Association between interruption of intervention and language performance in young children with language delay—a cohort study during COVID-19 pandemic

**DOI:** 10.3389/fped.2023.1240354

**Published:** 2023-09-15

**Authors:** Shao-Chih Hsu, Alice May-Kuen Wong

**Affiliations:** ^1^ Department of Physical Medicine and Rehabilitation, Chang Gung Memorial Hospital, Taoyuan, Taiwan; ^2^ Department of Physical Medicine and Rehabiltiation, New Taipei City Municipal Tucheng Hospital, Chang Gung Memorial Hospital, Tucheng branch, New Taipei City, Taiwan

**Keywords:** developmental language delay, language therapy, disruption of language intervention, language ability, COVID-19

## Abstract

**Introduction:**

To assess the association between a three-month interruption of language intervention programs and the language performance of children with language delay during the COVID-19 pandemic, and to identify which children are more vulnerable to such interruptions.

**Materials and methods:**

This is a retrospective study involving 33 children with language delay who experienced a three-month suspension of language interventions due to the COVID-19 pandemic. We collected their demographic data and language performance scores from the Comprehensive Developmental Inventory for Infants and Toddlers—Diagnostic test (CDIIT-DT) at four different time points. The scores were analyzed using a Wilcoxon Signed Ranks test.

**Results:**

The median scores of language comprehension and overall language ability showed a decreasing trend during the interruption period. However, resuming interventions post-interruption showed a statistically significant increase in all language domains. Children in the borderline delay group (CDIIT-DT DQ scores between 71 and 85) were more likely to experience a decline in their language abilities during the interruption.

**Discussion:**

This is the first study to reveal a decreasing trend in language performance during interruption periods, and highlighting the significance of post-interruption language interventions in facilitating improvements. Furthermore, our study brings attention to the heightened vulnerability of children exhibiting borderline language delay in overall language ability tests when faced with interruptions in language interventions.

## 1. Introduction

Language delay, a condition in which children fail to meet the expected developmental milestones for their age group in terms of language comprehension and/or expression (1, 2), is common in young children, affecting approximately 5% to 12% of those between the ages of 2 and 5 (3, 4). Early identification and intervention can prevent language delay from interfering with formal education and behavioral adjustment (5). There have been numerous studies conducted to identify risk factors, predictors, and prevalence of language disorders in children (3, 6–10). Additionally, there is a considerable body of research focused on interventions for children with language delay (11–13). Some studies have reported outcome predictors specifically related to children with delayed expressive language (14, 15). However, it is worth noting that there are currently no studies investigating the influence of interrupting interventions on these children. Ethical considerations have made it impractical to interrupt interventions for children with language delay for research purposes. During the COVID-19 pandemic, Taiwan suspended non-emergency medical treatments, including language interventions for three months. This event provided a unique opportunity to study this topic. Our study aimed to explore the relationship between intervention interruptions and language performance in young children with language delay, and to identify which groups of children are more vulnerable to such interruptions.

## 2. Materials and methods

### 2.1. Patient inclusion

This retrospective study analyzed 33 children who were diagnosed with language delay or borderline language delay using the Comprehensive Developmental Inventory for Infants and Toddlers—Diagnostic test (CDIIT-DT) (16–20) and underwent language intervention programs at New Taipei City Hospital Tu Cheng Branch between June 2020 and August 2022. All these children experienced a three-month interruption of the program from April 2021 to July 2021 due to a sharp surge in Covid-19 cases in Taiwan.

### 2.2. Data collection

We collected general data from each study child with language delay, including date of birth, gender, gestational age, birth weight, medical history, family history, multilingual speaking, parents' education level, main caregiver, siblings' condition, early childhood education condition, and other developmental delay conditions. We used the CDIIT-DT to evaluate the language performance of each study child. Raw scores were collected from three subdomains (language comprehension, language expression, and overall language ability) at four different time points: T1—the first assessment before interventions, T2—the last assessment before the three-month interruption, T3—the first assessment after the three-month interruption, and T4—the last assessment obtained from patients' medical records.

### 2.3. Comprehensive developmental inventory for infants and toddlers (CDIIT)

The CDIIT was created by a multidisciplinary team in Taiwan in 1995 to evaluate the developmental levels of infants and toddlers between 3 and 71 months old, encompassing seven age groups. The CDIIT includes both a diagnostic test (CDIIT-DT) and a screening test (CDIIT-ST). These components are used to assess five developmental areas: motor skills, language skills, cognition, social skills, and self-care skills.

The CDIIT utilizes age-related norms established for the Taiwan population, with a mean score of 100 and a standard deviation of 15. It has been shown to exhibit good reliability and validity in previous studies. The CDIIT is generally supported as a norm-referenced test for evaluating developmental changes of children with developmental delay and outcome measures of pediatric intervention programs (16–19).

### 2.4. Language intervention programs in Taiwan

In Taiwan, young children are taken to pediatric rehabilitation clinics when there is a suspicion of developmental delay. Caregivers or kindergarten teachers usually notice these delays. If a physician clinically assesses children and identifies a language delay, they are referred to a speech-language pathologist for diagnostic tests. Once the reports confirm a language developmental delay, language intervention programs begin for them once per week.

In the Physical Medicine and Rehabilitation Department of New Taipei City Hospital Tu Cheng Hospital, a single pediatric speech-language pathologist utilized CDIIT-DT to track the language performance of children with language delay during intervention programs every three to six months. The intervention programs were discontinued when the CDIIT-DT indicated that the language delay was no longer present. Subsequently, every six months to a year, the speech-language pathologist utilized CDIIT-ST to track the language development of this children until they reached the age of six.

### 2.5. Statistical analysis

In this study, we transformed the raw scores into developmental quotient (DQ) scores and utilized the Wilcoxon Signed Ranks test (significance level: *p* < 0.05) to analyze the differences between various time points. The “W” test statistic in the Wilcoxon Signed Ranks test represents the sum of ranks computed from the absolute differences between paired observations, facilitating the assessment of significant variations between these paired measurements.

To further investigate the CDIIT score regression following intervention interruption, we segregated the children into two groups based on this specific criterion. To examine the potential correlations between each variable and CDIIT score regression, univariate logistic regression and multivariate logistic regression were employed (significance level: *p* < 0.05). Moreover, for the construction of a prediction model, we applied machine learning algorithms, including KNN, decision tree, and random forest. All analyses were conducted using Python 3, capitalizing on its extensive libraries, and figures were generated using both Python 3 and Prism 9 software.

With regard to ethical considerations, the study was reviewed and approved by the Institutional Review Board of Chang Gung Memorial Hospital, with a waiver of informed consent granted for the use of de-identified data from routine clinical care, ensuring ethical compliance. This study followed the STROBE reporting guideline.

## 3. Results

### 3.1. Findings on language comprehension, expression, and overall language ability

Of all the children studied, 19 were male and 14 were female. Characteristics of Study Participants were presented in the Table 1. The median scores of language comprehension, language expression, and overall language ability at different time points are presented in Table 2. Our findings showed that the scores for language comprehension were consistently higher than those for language expression across all time points. Compared with T3, there were statistically significant increases of scores at T4 among all three domains: language comprehension (*W* = 315, *p* = 0.0001), language expression (*W* = 140, *p* = 0.04), and overall language ability (*W* = 228, *p* = 0.001).

**Table 1:** Characteristics of study participants and comparison of Two groups based on regression Status after intervention interruption.

	CDIIT score regression after interruption	No CDIIT score regression after interruption	*p*-value
Patients (*n*)	15	18	
Age (years) (mean ± standard deviation)	3.6 ± 1.5	3.6 ± 1.4	0.96
Sex			0.25
Male	7	12	
Female	8	6	
Gestational age			0.99
>37 weeks	15	15	
<37 weeks	0	3	
Birth body weight			
>2,500 g	13	16	0.85
<2,500 g	2	2	
Medical history			0.70
Nil	9	10	
Attention deficit hyperactivity disorder	5	6	
Autism	1	2	
Multilingual family			0.40
1 language	14	15	
>1 language	1	3	
Main caregiver			0.26
Parents	11	16	
Other than parents	4	2	
Having older siblings (age difference <3 years)			0.41
Yes	8	11	
No	7	7	
Preschool education (3 K, Pre-K, K)			0.52
Yes	10	10	
No	5	8	
Co-occurring Developmental Delays			0.28
Nil	4	5	
Gross motor (GM)	2	0	
Fine motor (FM)	1	0	
GM + FM	3	3	
GM + FM + Cognition	5	7	
Cognition	0	3	
Additional Rehabilitation Programs			0.51
Nil	2	4	
Physical therapy (PT)	1	0	
Occupational therapy (OT)	4	7	
PT + OT	8	7	

T3 represents the first assessment conducted after the three-month interruption. Statistical significance is denoted by *p* < 0.05.

**Table 2:** Median CDIIT-DT scores and significance levels at different timepoints across language comprehension, expression, and ability domains.

		T1	T2	T3	T4
Language Comprehension	25 percentiles	64.5	66.0	54.0	69.5
	Median	71.0	77.0	74.0	79.0
	75 percentiles	83.0	83.5	82.0	89.5
*p*-value	vs. T1	N/A	0.27	0.81	0.005
	vs. T2	N/A	N/A	0.10	0.04
	vs. T3	N/A	N/A	N/A	0.0001
Language Expression	25 percentiles	54.0	54.0	63.0	58.0
	Median	71.0	69.0	70.0	77.0
	75 percentiles	78.5	79.0	78.0	90.0
*p*-value	vs. T1	N/A	0.79	0.40	0.07
	vs. T2	N/A	N/A	0.46	0.02
	vs. T3	N/A	N/A	N/A	0.04
Overall Language Ability	25 percentiles	54.0	54.0	54.0	60.5
	Median	71.0	72.0	71.0	75.0
	75 percentiles	76.5	76.0	77.0	90.5
*p*-value	vs. T1	N/A	0.59	0.91	0.01
	vs. T2	N/A	N/A	0.55	0.006
	vs. T3	N/A	N/A	N/A	0.001

vs., versus. T1: the first assessment before interventions, T2: the last assessment before the 3-month interruption, T3: the first assessment after the 3-month interruption, T4: the last assessment obtained from patients’ medical records.

### 3.2. Trends in CDIIT-DT DQ scores and the impact of intervention interruption

**Figure 1:**
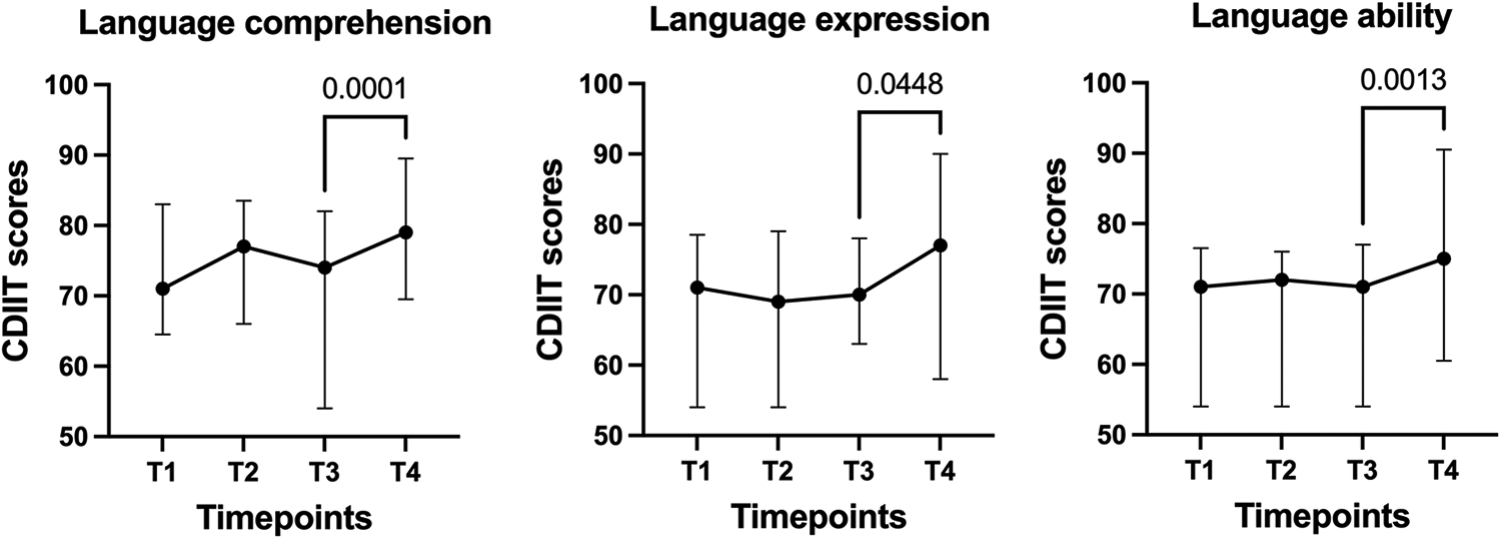
Trends in CDIIT-DT DQ scores over time. T1: the first assessment before interventions, T2: the last assessment before the 3-month interruption, T3: the first assessment after the 3-month interruption, T4: the last assessment obtained from patients’ medical records. The plot shows the median and interquartile range of CDIIT-DT DQ scores at each timepoint.

Figure 1 illustrates the trends in CDIIT-DT DQ scores over time for the domains of language comprehension, language expression, and overall language ability. Comprising the three line charts, the trend of language comprehension aligns consistently and synchronously with the overall language ability. The median scores of the two domains, language comprehension and overall language ability, showed a decreasing trend during the interruption. Moreover, resuming the intervention after the interruption showed a statistically significant increase in all language domains.

### 3.3. Analysis of individual variable contributions to CDIIT-DT DQ score regression in children with language delay following intervention interruption

To identify key variables that might contribute to CDIIT score regression following intervention interruption, we conducted both univariate logistic regression and multivariate logistic regression analyses. However, none of the variables exhibited statistical significance. The *p*-values obtained from the univariate logistic regression analysis are detailed in Table 1.

### 3.4. Identifying vulnerable groups: language ability scores and therapy interruption

**Figure 2:**
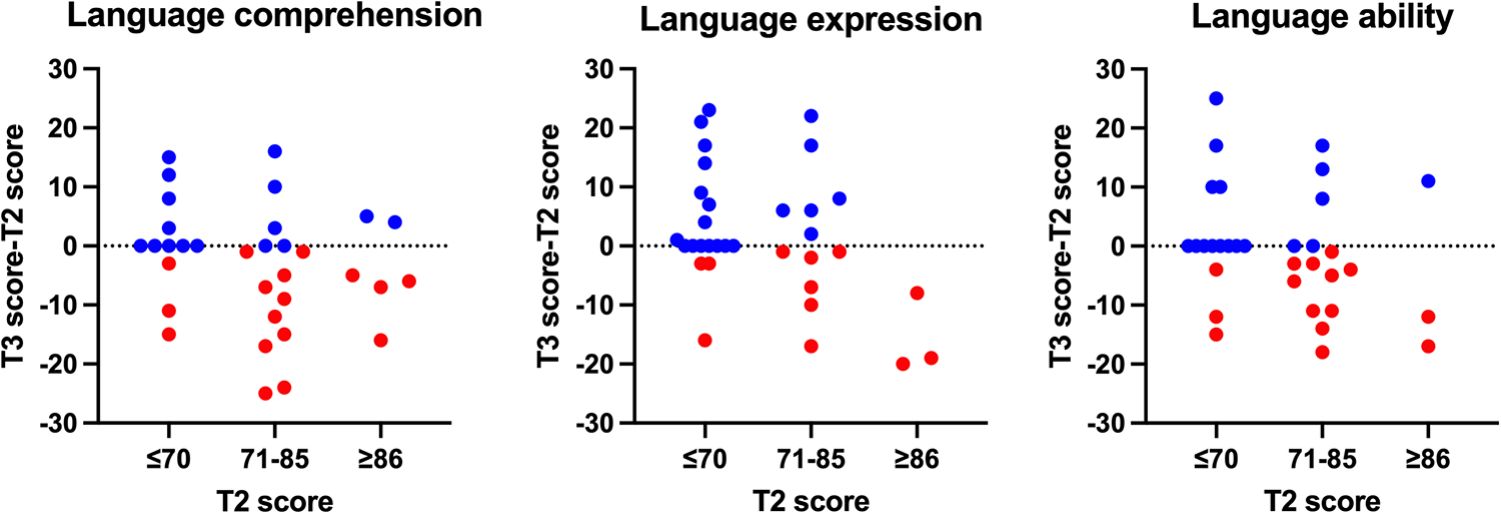
Scatter plot of CDIIT-DT DQ scores and T3-T2 score differences. X-axis: CDIIT-DT DQ score (interval: 10 points), Y-axis: T3-T2 score difference, T2 score: score evaluated at last assessment before the 3-month interruption, T3 score: score evaluated at first assessment after the 3-month of interruption. The red dots represent cases where the CDIIT-DT DQ score at T3 is lower than the score at T2, while the blue dots indicate cases where the CDIIT-DT DQ score at T3 is higher than or equal to the score at T2.

To identify which group were more vulnerable to speech therapy interruption, we divided studied children based on their CDIIT-DT DQ scores at T2. We used 15 points as an interval to group these children. We observed a group of children with overall language ability scores ranging from 71 to 85 (borderline delay) at T2. Out of the 15 children in this group, 10 showed a decline in scores between T2 and T3, while 2 maintained stable scores (Figure 2).

### 3.5. Constructing a prediction model for CDIIT-DT DQ score regression following intervention interruption

**Figure 3:**
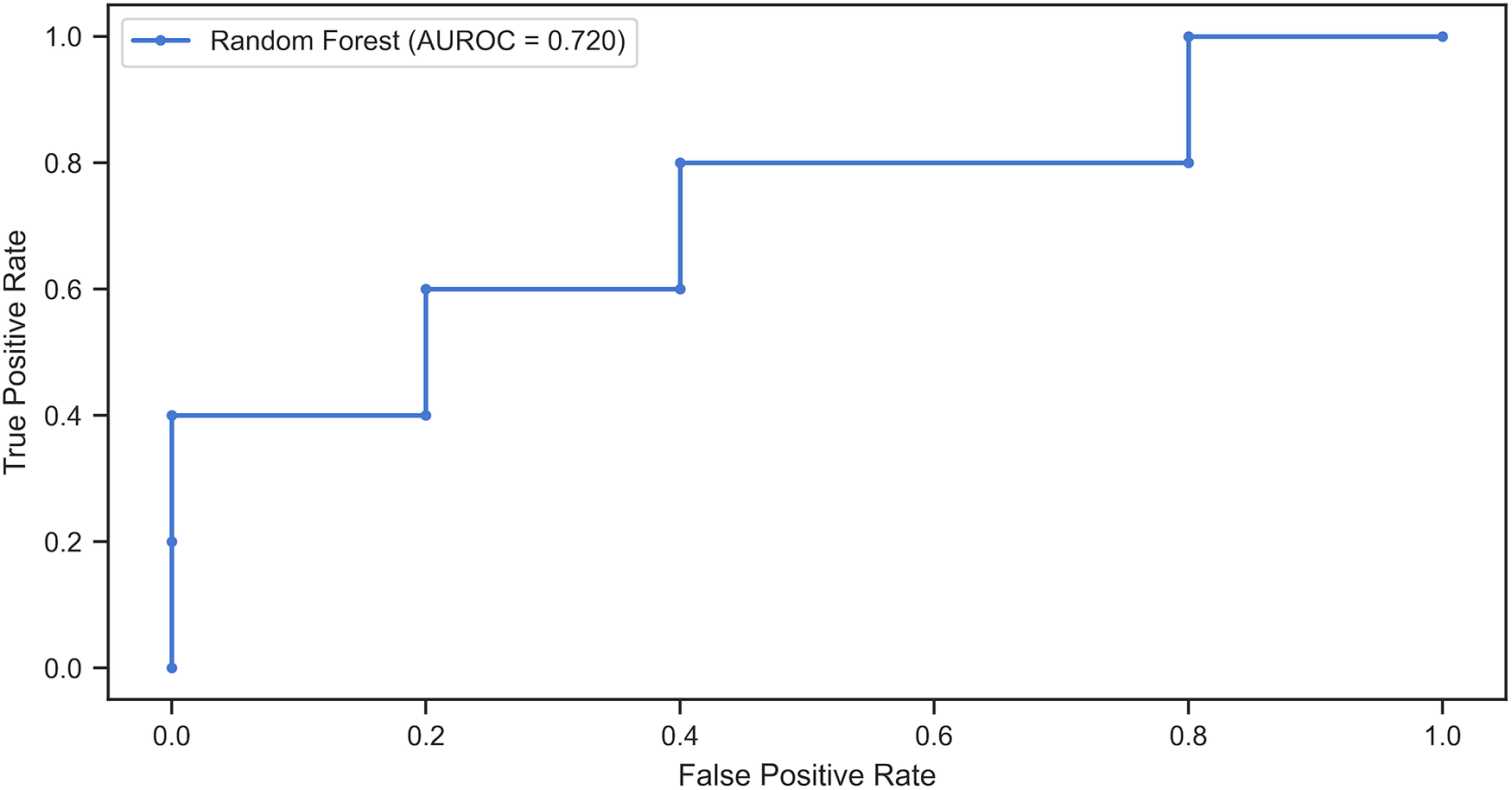
Receiver operating characteristic (ROC) curve of random forest algorithm for predicting CDIIT-DT score regression after intervention interruption. The ROC curve illustrates the performance of the Random Forest algorithm in predicting CDIIT-DT score regression after intervention interruption. The Area Under the ROC Curve (AUROC) was calculated to be 0.720, indicating a moderate level of predictive accuracy.

While no key variable was identified as contributing to CDIIT score regression after intervention interruption, we endeavored to construct a prediction model utilizing all available variables. By employing machine learning algorithms such as KNN, decision tree, and random forest, we sought to find the most effective predictive approach. The random forest algorithm emerged as the most accurate, achieving a 70% accuracy rate and an area under the ROC curve of 0.720 (Figure 3).

## 4. Discussion

This study is the first to examine the effects of interrupting language intervention programs on children with language delay, which occurred during the COVID-19 outbreak in Taiwan in 2021. The unique circumstances presented by the pandemic provided an opportunity to investigate this topic. While it is unlikely that similar situations will arise in the future, the findings of this study remain valuable in understanding the consequences of unexpected disruptions in healthcare services. Despite the numerous studies conducted during the global COVID-19 pandemic focusing on strategies and alternative language intervention methods, such as telepractice speech therapy (21–23), little is known about the specific impact of pandemic-induced intervention interruption on children with language delays. Hackenberg et al. reported a high psychosocial burden experienced by parents of children with speech and language disorders due to therapy pause during the Covid-19 pandemic; however, the effects on their children's speech and language abilities were not addressed (24).

Regarding the relationship between intervention interruption and language performance in children with language delays, our findings indicated a decreasing trend during the interruption period, while post-interruption language interventions were significantly associated with performance improvements. These findings can provide clinicians and parents with an overview of language performance trends following rehabilitation interruption and encourage them to pursue post-interruption interventions. In addition, Hackenberg el. reported parents of children with speech and language disorders had more fears and worries about their children's development (24). In a clinical setting, our study can alleviate parents' psychosocial burden and increase parental compliance and confidence in resuming intervention post-interruption.

Children with developmental delay typically demonstrate stronger language comprehension abilities relative to their language expression skills (25). This observation aligns with our results, which showed that the mean CDIIT-DT scores for language comprehension consistently exceeded those for language expression at all evaluated time points. More notably, language comprehension scores exhibited greater sensitivity to the impact of continuity and discontinuity in rehabilitation programs compared to language expression scores. In our study, the slopes in the line chart depicting language abilities closely paralleled those for language comprehension (Figure 1). These results reinforce the substantial role that language comprehension plays in the speech and language abilities of children. Earlier studies have likewise reported that language comprehension could serve as a reliable predictor of language expression outcomes (3). Therefore, assessing language comprehension is not just crucial for differential diagnosis, but also invaluable for evaluating outcomes in children with speech and language disorders.

To further enhance language comprehension in children with developmental delay, several strategies can be employed (26). These include Enhanced Milieu Teaching (EMT), a conversation-based therapy technique that uses the child's interests as opportunities to model and prompt language use in everyday contexts. Parent-based Video Home Training, where parents are trained in attachment, referencing, relevance, and connectivity of language, can also be beneficial. Techniques such as pausing and expanding in shared book reading and in everyday situations can be used to encourage children to choose or initiate a topic of interest to them. Interactive book reading with expository books and language facilitation strategies can also be employed to focus children's attention on the expository structure and help them construct responses to questions. It is possible that tailoring post-interruption intervention strategies to enhance comprehension could expedite improvements in expressive abilities in overall language abilities.

Our study is also the first to identify which groups of children are more vulnerable to interruptions in language interventions. Our results revealed that among the children with overall language ability scores ranging from 71 to 85 at T2, 10 out of the 15 children in this group exhibited a decline in scores between T2 and T3. Similar results were observed in the language comprehension test (Figure 2). In clinical practice, parents often assume that their child is approaching age-appropriate milestones, overlooking the importance of language intervention during the borderline delay phase. However, our study highlights the particular importance of maintaining ongoing language intervention during this phase, as discontinuation poses a high risk of score regression. Further research is necessary to corroborate this finding, which would provide clinicians and parents with a better understanding of the optimal timing for ceasing interventions.

Although this study is the only one examining young children with language delay who experience interruptions in language intervention programs, there were some limitations to this study. The most notable constraint is the small sample size, which may have curtailed the statistical power of our analyses and led to an absence of statistically significant findings in both univariate and multivariate logistic regression. This small sample size could explain our inability to detect meaningful differences. Nevertheless, our application of a machine learning algorithm enabled us to predict CDIIT score regression after intervention interruption with an accuracy of 70%. We anticipate that expanding the database for model training could enhance this accuracy further. It is essential to note that our findings are preliminary and derived from a small, heterogeneous sample. These initial results emphasize the need for larger and more diverse studies to corroborate and solidify our conclusions.

## Data Availability

The raw data supporting the conclusions of this article will be made available by the authors, without undue reservation.
